# Data demonstrating the challenges of determining the kinetic parameters of P-gp mediated transport of low-water soluble substrates

**DOI:** 10.1016/j.dib.2017.11.092

**Published:** 2017-12-06

**Authors:** Burak Ozgür, Lasse Saaby, Kristine Langthaler, Birger Brodin

**Affiliations:** a Section of Pharmaceutical Design and Drug Delivery, Department of Pharmacy, University of Copenhagen, Universitetsparken 2, DK-2100 Copenhagen, Denmark; b Bioneer-FARMA, Department of Pharmacy, University of Copenhagen, Universitetsparken 2, DK-2100 Copenhagen, Denmark; c H. Lundbeck A/S, Ottiliavej 9, 2500 Valby, Denmark

## Abstract

The presented data are related to the research article entitled “Characterization of the IPEC-J2 MDR1 (iP-gp) cell line as a tool for identification of P-gp substrates” by Ozgur et al. (2017) [1]. This data report describes the challenges of investigating the concentration-dependent transport of P-glycoprotein (P-gp) substrates with relatively low aqueous solubility. Thus, we provide solubility data on two prototypical P-gp substrates, digoxin and rhodamine 123, representing P-gp substrates with a relatively low- and high-aqueous solubility, respectively. We present a hypothetical Michaelis-Menten curve of the P-gp mediated transport of digoxin to demonstrate that the maximal donor concentration, which can be reached in the experimental transport buffer, is too low to yield transport data in the saturable range of the Michaelis-Menten relationship. Furthermore, we present data on the bidirectional transport of digoxin and rhodamine 123 across cell monolayers of the MDCK II MDR1 cell line and iP-pg cell line in the presence of the selective P-gp inhibitor, zosuquidar/LY335979.

**Specifications Table**

**Table:** 

Subject area	*Pharmaceutic*
More specific subject area	*Efflux transporter, drug delivery*
Type of data	*Table, Figures*
How data was acquired	*HPLC-system consisting of an UltiMate 3000 pump (Thermo Fisher Scientific, Copenhagen, Denmark), a Kinetex column XB-C18 particle size 5 μm, 4.6 x 100 mm (Phenomenex, Torrance, CA, USA) and a UV-detector (Waltham, MA, USA).*
	*A fluorescence microplate reader (NOVOstar Microplate Reader, BMG Labtech, Offenburg, Germany).*
	*A liquid scintillation analyzer (Packard Tri-Carb 2100 TR; Canberra, Dreich, Germany).*
	*GraphPad Prism Software (version 7.0, Inc. San Diego, CA, USA).*
Data format	*Raw and analyzed*
Experimental factors	*N/A*
Experimental features	*The apparent solubility of both digoxin and rhodamine 123 was estimated thermodynamically by adding the experimental buffer systems directly to solid crystalline digoxin or rhodamine 123.*
	*Transport data were generated by measuring fluxes of digoxin and rhodamine 123 across cell monolayers of the MDCK II MDR1 and iP-gp cell lines, for further details see**[1]*.
Data source location	*University of Copenhagen, Denmark*
Data accessibility	*Data are available with this article*

**Value of the data**

• Data present the aqueous solubility of two prototypical P-gp substrates, digoxin and rhodamine 123. These data can be used for comparisons with solubilities obtained in other buffer systems, and to guide researchers working with these compounds in cell based assays.
• Data provide measurements of solubilities of digoxin and rhodamine 123 in the absence and presence of bovine serum albumin. These values show protein binding in the near-solubility range and are useful in experimental protocol design.
• Data provide a comparison of the passive permeability of the two P-gp substrates, [^3^H]-digoxin and rhodamine 123, across cell monolayers of P-gp expressing cell lines. The data can be used for modeling of membrane permeation of lipophilic substrates of P-gp, and for baseline permeability comparisons between barrier cell models.

## 1 Data

**Fig. 1:**
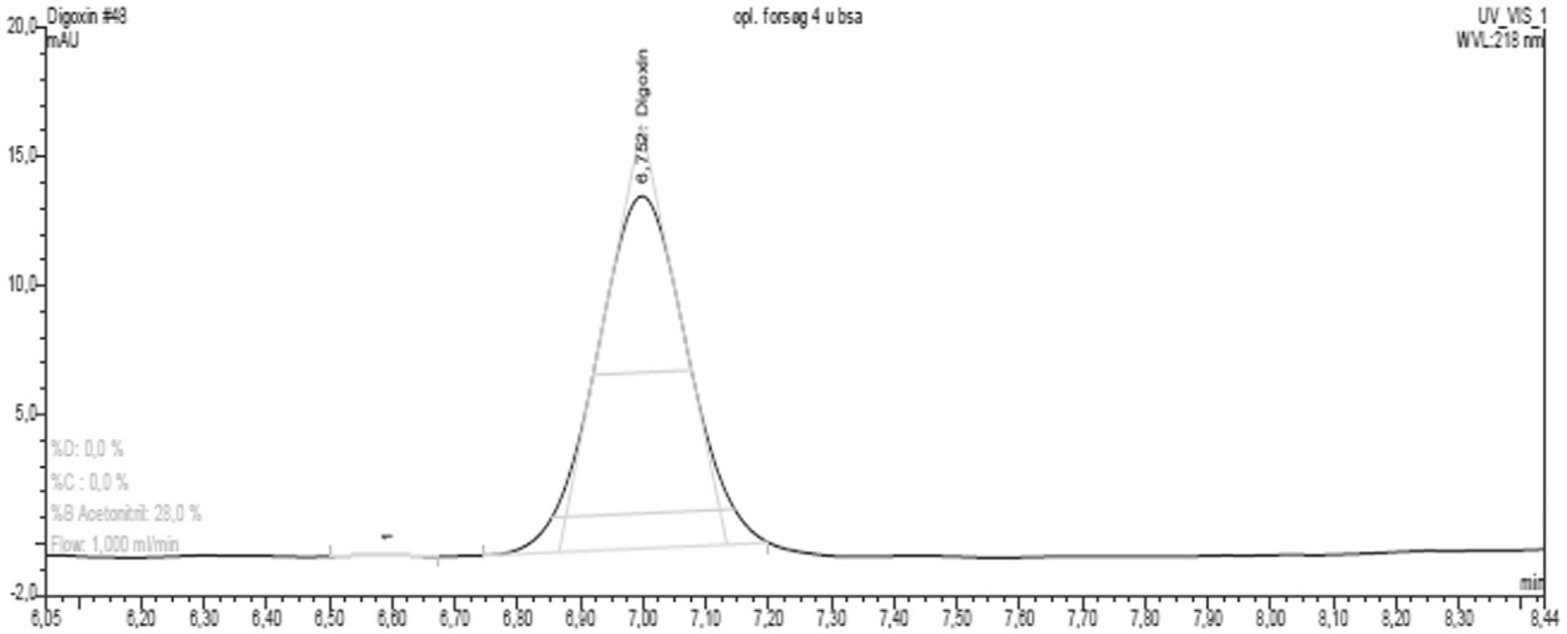
A representative HPLC-UV chromatogram of digoxin.

**Fig. 2:**
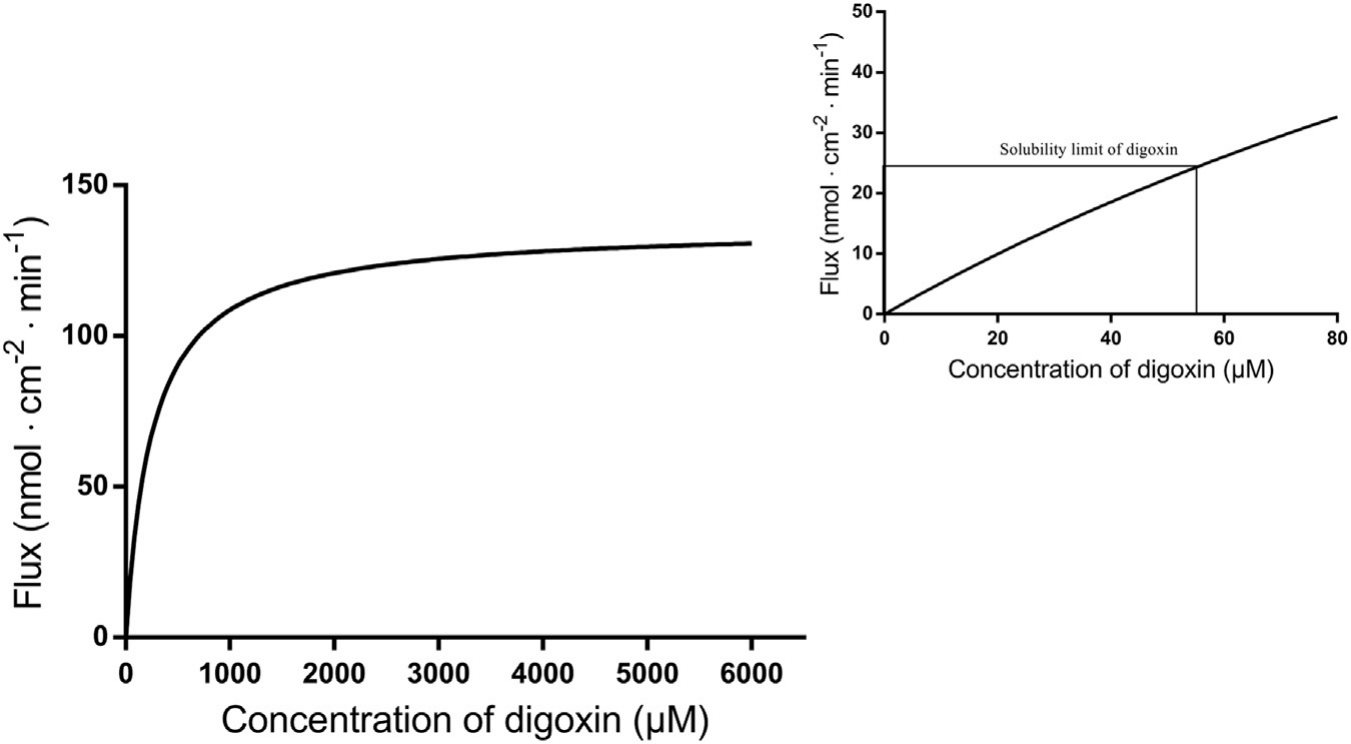
Illustration of a hypothetical Michaelis-Menten curve of the P-gp mediated transport of digoxin. The box in the right figure shows the solubility limit of digoxin in the transport buffer (HBSS^+^) supplemented with 2% (v/v) DMSO.

**Fig. 3:**
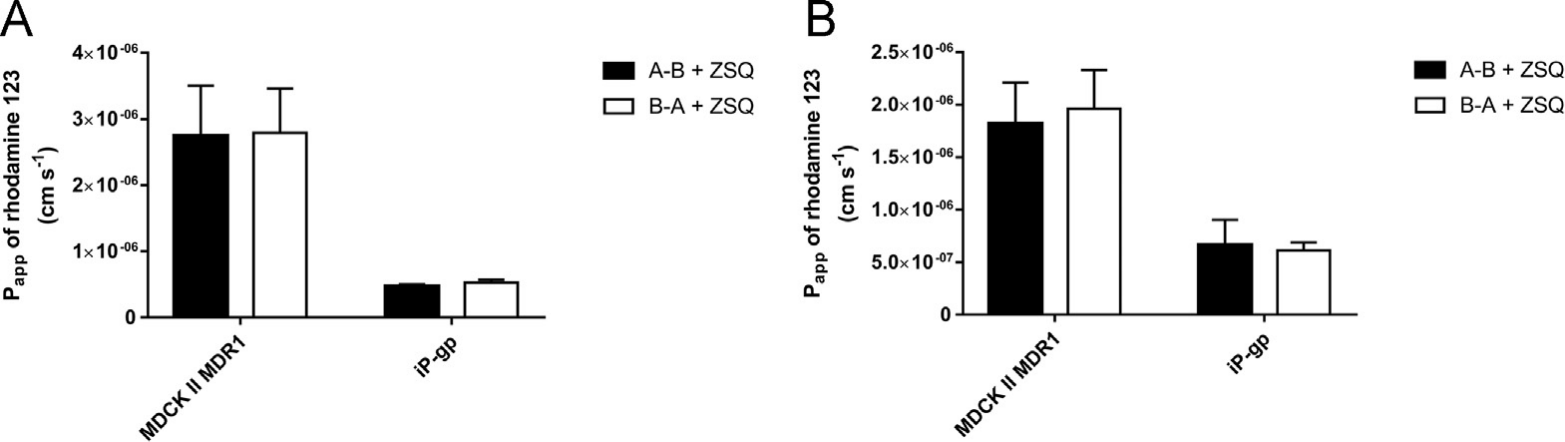
Bidirectional transport of [^3^H]-digoxin and rhodamine 123 across MDCK II MDR1 and iP-gp cell monolayers in the presence of 2 µM zosuquidar (ZSQ). The apparent permeability P_app_ values were calculated from the steady state fluxes. The filled bars illustrate P_app,A-B_ values, whereas open bars illustrate P_app,B-A_. Values are presented as mean±SEM of three individual passages of three individual permeable supports (*n*=3, total *N*=9).

This brief data article provides solubility data on two prototypical P-gp substrates, digoxin and rhodamine 123, in aqueous buffer systems (Table 1). Fig. 1 shows a representative HPLC chromatogram of digoxin. Fig. 2 shows a hypothetical Michaelis-Menten saturation curve of the P-gp mediated transport of digoxin. Fig. 3 shows data on bidirectional transport of [^3^H]-digoxin and rhodamine 123 across cell monolayers of the MDCK II MDR1 and iP-gp cell lines in the presence of the selective P-gp inhibitor, zosuquidar.

**Table 1:** The apparent solubility (S_app_​) of digoxin and rhodamine 123 in HBSS^+^ and HBSS^−^, supplemented with 2% DMSO. Solubility data are shown as means±SD of one individual experiments of triplicates (*n*=1, total *N*=3).

Compound	S_app_ in HBSS^+^ + 2% DMSO (µM)	S_app_ in HBSS^−^ + 2% DMSO (µM)
Digoxin	55.0±5.7	42.9±5.4
Rhodamine 123	1088±13	1053±20

## 2 Experimental design, materials and methods

### 2.1 Solubility measurements of digoxin and rhodamine 123 in transport buffer

The apparent aqueous solubility (S_app_) of digoxin and rhodamine 123 was estimated in the experimental transport buffer as described previously [1]. Briefly, the buffer systems were Hank's balanced salt solution (HBSS) supplemented with 10 mM HEPES (pH 7.4), 0.375% (v/v) sodium carbonate, and finally with or without 0.05% BSA. The buffer systems with and without 0.05% BSA are abbreviated HBSS^+^ and HBSS^−^, respectively. The buffer systems were supplemented with 2% (v/v) DMSO, unless otherwise stated.

### 2.2 The concentration dependent transport of digoxin

In the referred article, the apparent transport of digoxin was determined at a concentration range of 0.0251–13 µM across the iP-gp cell monolayers cultured on permeable supports (1.12 cm^2^) in the basolateral to apical direction [1]. Experiments were performed in the absence and presence of 2 µM zosuquidar to estimate the total and the passive transport, respectively [1]. By subtracting the passive transport from the total transport at the respective substrate concentrations, the P-gp mediated transport was obtained. The fluxes of the P-gp mediated transport versus donor concentrations of digoxin were fitted into the Michaelis-Menten equation, and the kinetic parameters *K*_m_ and *V*_max_ were derived to 253 µM and 136 nmol cm^-2^ min^-1^, respectively. Based on these kinetic parameters, a hypothetical Michaelis-Menten relationship of the P-gp mediated transport was drawn using GraphPad Prism (Fig. 2).

### 2.3 Bidirectional transport of [^3^H]-digoxin and rhodamine 123

The bidirectional transport experiments were performed as described previously, Ref: [1]. Briefly, the fluxes of [^3^H]-digoxin and rhodamine 123 were investigated across cell monolayers of the MDCK II MDR1 and iP-gp cell lines in the presence of 2 µM zosuquidar. Fig. 3 shows the apparent permeabilities (P_app_) in the basolateral to apical direction (B-A) and apical to basolateral direction (A-B). The donor concentrations of [^3^H]-digoxin and rhodamine 123 were 0.0251 µM and 3.12 µM, respectively. HBSS^+^ with 0.5% DMSO was used as the transport buffer (For further details see [1]).

## References

[1] Ozgur B., Saaby L., Langthaler K., Brodin B. (2017). Characterization of the IPEC-J2 MDR1 (iP-gp) cell line as a tool for identification of P-gp substrates. J. Pharm. Sci..

