# Supplementary material for: Data demonstrating the challenges of determining the kinetic parameters of P-gp mediated transport of low-water soluble substrates
Source: Data Brief. 2017 Dec 6;16:655–9. doi: 10.1016/j.dib.2017.11.092 (PMC5848149; doi:10.1016/j.dib.2017.11.092)
Supplement: Supplementary file 1 — Supplementary material [file mmc1.docx]

Conflict of interest:

Lasse Saaby is employed by the company Bioneer-Farma, which offers commercial screening of drug compounds using the iPgp cell line. The cell line is available from Birger Brodin for academic studies, upon request.
